# Supplementary material for: Target the Heart: A New Axis of Alzheimer’s Disease Prevention
Source: J Dement Alzheimers Dis. Author manuscript; Available in PMC 2025 Sep 9. (PMC12416075; doi:10.3390/jdad2020010)
Supplement: Supplementary Figure 1 [file NIHMS2099309-supplement-Supplementary_Figure_1.pdf]

Treatment	Difference in Activity (Treated-DMSO)					Difference in Vision (Treated-DMSO)					Scoot (3-20p)		Burst (>20p)		% Edge		Clockwise (1h, Vis)		Upward Orientation with Visual Stimuli					Turn Angle	
	1hr	P15	Hab	S	E	R	G	B	FR	RGB	Sc-1h	Sc-V	Bu-1h	Bu-V	Ed-1h	Ed-V	Cw-1h	Cw-V	Or-R	Or-G	Or-B	Or-FR	Or-RGB	Turn-1h	Tabs-1hr
DMSO	0.0	0.0	0.0	0.0	0.0	0.0	0.0	0.0	0.0	0.0	0.0	0.0	0.0	0.0	0.0	0.0	0.0	0.0	0.0	0.0	0.0	0.0	0.0	0.0	0.0
5 uM Cyclosporine	3.0	**16.8**	**-9.2**	-2.1	**14.1**	**-16.6**	**-11.7**	-6.8	-1.6	**-9.2**	6.5	**10.7**	-3.5	-2.5	**-11.1**	-4.7	-2.3	0.7	-3.7	-1.8	-3.7	-3.7	-3.2	-1.0	-2.9
10 uM Cyclosporine	**12.0**	**14.3**	**-6.8**	-1.8	5.9	**-10.8**	-3.1	-5.4	**-9.1**	**-7.1**	**7.3**	**12.0**	**4.7**	**11.7**	**10.6**	**13.5**	-0.8	-2.6	-4.4	-0.5	-2.9	-2.4	-2.6	-0.4	**3.7**
10 uM Nebivolol	5.4	**9.5**	-4.9	-2.3	**12.2**	-7.7	-6.0	-8.1	**-11.7**	**-8.4**	**9.7**	**7.1**	**-4.2**	**6.1**	**12.0**	**13.2**	-5.4	-0.2	**-29.8**	**-14.1**	**-20.6**	**-38.9**	**-25.8**	-0.8	**-3.6**
5 uM Nebivolol + 5 uM Cyclosporine	**18.0**	**24.4**	**-10.9**	-3.9	**23.1**	**-16.7**	-4.7	-15.2	-6.9	**-10.8**	**17.5**	**19.8**	0.5	3.0	-9.7	3.4	1.7	4.2	-4.6	-5.2	**-9.7**	**-19.3**	**-9.7**	0.2	0.6
10 uM Losartan	**17.5**	12.7	-7.8	1.0	-4.4	-2.9	13.8	7.1	3.5	5.4	**9.8**	**8.9**	7.7	10.7	8.5	**13.1**	5.6	4.8	8.5	8.0	7.2	8.3	8.0	1.8	6.4
5 uM Losartan + 5 uM Cyclosporine	14.8	12.2	-2.1	6.4	-4.5	7.4	1.5	4.9	5.2	4.8	8.4	4.8	6.4	6.9	5.2	**10.5**	-0.1	-8.1	5.8	4.7	11.0	13.1	8.7	-1.3	6.4
10 uM Carvedilol	2.1	4.6	-9.5	3.3	5.0	7.5	6.6	1.5	2.9	4.6	**8.4**	**9.2**	**-6.3**	2.4	-23.0	1.7	-0.6	3.3	-13.0	4.5	-13.3	-12.0	-8.4	-0.6	**-4.1**
5 uM Carvedilol + 5 uM Cyclosporine	1.8	1.8	-9.2	8.5	8.6	0.4	-0.1	3.0	20.9	6.1	6.7	**9.2**	-4.9	3.9	-12.8	3.4	3.6	-2.1	-4.5	-13.4	-8.9	-10.1	-9.2	-0.2	-2.6
10 uM Doxazosin	**9.1**	2.9	-5.9	3.2	**20.8**	-7.2	-6.2	-1.3	-4.8	-4.9	**9.1**	**7.3**	0.0	**9.2**	-15.7	4.9	6.0	1.5	-9.4	-2.6	-3.5	1.8	-3.4	0.1	3.3
5 uM Doxazosin + 5 uM Cyclosporine	**16.5**	10.3	**-9.4**	0.6	**18.3**	-5.8	-0.8	-7.3	-3.0	-4.2	**12.2**	**9.8**	4.3	**12.8**	**-19.2**	-3.3	-1.6	-5.0	-10.4	-0.3	0.9	3.0	-1.6	-0.6	**7.2**
10 uM Irbesartan	**22.0**	13.1	-2.7	-2.8	2.6	0.8	6.0	-3.4	-2.6	0.2	**9.8**	**8.3**	**12.2**	**9.5**	**7.4**	**10.8**	-1.8	-2.0	2.2	4.6	3.1	2.9	3.2	0.1	**11.5**
5 uM Irbesartan + 5 uM Cyclosporine	**31.2**	**17.9**	**-9.1**	3.3	6.7	-6.8	-1.1	-3.4	-4.8	-4.0	**8.9**	**7.6**	**22.3**	**19.7**	**11.8**	**13.2**	-2.0	-7.1	-3.1	4.0	5.9	5.3	3.0	-1.6	**18.2**
10 uM Prazosin	8.0	4.3	-1.6	4.0	6.8	3.2	5.2	3.1	1.3	3.2	6.7	**9.7**	1.3	**8.5**	-15.0	1.8	6.5	2.3	-5.2	0.0	-3.6	4.9	-1.0	0.4	1.4
5 uM Prazosin + 5 uM Cyclosporine	**15.2**	10.9	3.7	0.2	0.2	-5.9	2.8	6.2	-7.2	-1.0	**9.4**	**8.4**	5.7	**14.6**	-6.4	5.9	-0.1	1.2	-6.9	3.6	6.9	0.9	1.3	-0.7	5.2
10 uM Cilostazol	**22.4**	4.4	-0.2	0.2	-2.3	3.9	-3.9	-2.2	1.0	-0.3	7.3	**8.1**	15.1	10.8	**11.1**	**13.4**	-4.5	-6.9	-7.3	5.9	7.3	6.9	3.3	0.4	14.6
5 uM Cilostazol + 5 uM Cyclosporine	**26.4**	**25.7**	-5.5	-9.2	1.0	5.8	-1.6	-0.2	-3.4	0.1	**9.9**	**9.3**	**16.4**	**17.4**	**13.8**	**14.6**	-3.7	-2.7	6.5	8.5	-1.4	9.4	5.3	-1.3	**17.0**
10 uM Calcifediol	**20.2**	9.6	-3.4	**-15.6**	-0.4	-2.6	2.0	-1.5	8.6	1.6	2.1	**5.4**	**18.2**	**21.5**	**15.3**	**14.0**	-3.1	0.3	-3.6	2.4	-3.0	8.0	1.0	-2.3	**13.5**
5 uM Calcifediol + 5 uM Cyclosporine	**28.8**	12.5	**-10.9**	-8.2	-13.7	-2.8	-6.9	-5.9	-14.0	-7.4	6.5	1.7	**22.3**	**26.4**	**13.2**	**14.6**	-10.4	-5.5	1.5	-5.7	-6.5	-4.0	-3.7	0.0	**16.8**
10 uM Droperidol	**30.0**	**16.4**	-3.4	-2.9	**16.3**	2.2	-8.5	-6.6	-8.0	-5.2	**9.8**	**8.7**	**20.1**	**22.1**	-3.4	**11.4**	-0.2	-0.8	-14.3	-7.9	-3.4	-9.3	-9.0	1.6	**22.0**
5 uM Droperidol + 5 uM Cyclosporine	**24.9**	**22.6**	**-11.1**	1.9	**18.2**	-10.0	-1.3	-9.5	-0.1	-5.2	**12.0**	**12.7**	**13.0**	**17.9**	**10.3**	**15.7**	-2.1	4.9	-9.3	-18.7	-4.7	-7.9	**-10.8**	0.1	**13.5**
10 uM Eprosartan	**20.5**	-2.4	-3.1	7.0	-12.5	-9.3	1.8	-2.4	3.2	-1.7	9.5	**8.5**	11.1	**11.6**	**11.5**	**14.1**	1.0	-0.5	-0.9	-5.2	1.2	8.6	0.9	1.8	9.9
5 uM Eprosartan + 5 uM Cyclosporine	**38.2**	16.6	5.3	0.8	-15.8	-9.8	3.3	-1.9	-6.0	-3.6	7.4	4.1	**30.9**	**22.9**	**13.3**	**12.4**	3.1	-0.1	5.2	6.1	0.5	10.4	5.5	0.1	**27.1**
10 uM Telmisartan	6.3	5.2	4.3	1.5	2.2	17.4	2.2	**21.5**	19.4	**15.1**	1.6	**13.2**	4.7	8.8	7.6	**12.9**	2.2	3.1	8.7	2.6	14.5	23.4	12.3	0.0	2.2
5 uM Telmisartan + 5 uM Cyclosporine	9.4	13.2	-9.5	**-9.7**	6.6	12.2	-2.7	4.0	6.2	5.0	2.8	2.7	6.6	12.6	7.8	**13.5**	-2.0	-1.3	14.2	-0.8	11.9	12.5	9.4	-0.6	6.7
10 uM Trifluoperazine	-2.7	-9.4	-6.6	-4.0	-4.3	1.5	-2.1	-8.8	-4.8	-3.5	-1.7	**7.1**	-1.0	8.5	-2.0	**10.7**	-4.7	-3.7	-1.5	6.6	-0.3	7.0	3.0	-2.5	-0.5
5 uM Trifluoperazine + 5 uM Cyclosporine	-10.3	-13.6	-6.8	2.0	-8.6	-6.5	-6.0	-8.8	-9.9	-7.8	-6.4	3.4	-3.8	11.5	-0.8	10.2	-0.4	-5.8	-2.3	-6.2	1.5	4.7	-0.3	-0.9	-2.8
10 uM Mirtazapine	-3.1	-2.8	-8.4	-14.9	-4.0	-1.7	-2.5	-6.4	**-23.8**	-8.6	-4.4	4.8	1.3	**14.1**	3.0	**8.7**	-2.7	-3.3	-1.7	-2.7	-0.5	-2.8	-2.5	-1.5	1.8
5 uM Mirtazapine + 5 uM Cyclosporine	**-16.0**	-5.5	-5.6	-5.1	-3.4	6.3	-4.8	4.6	-18.0	-3.0	**-9.8**	**-8.5**	**-6.2**	2.1	**11.5**	**11.2**	-7.1	0.8	0.5	-1.8	5.9	6.2	2.7	-0.5	**-5.3**
10 uM Simvastatin	**6.5**	**10.3**	**-11.4**	-1.7	**26.5**	-3.9	-3.1	-4.1	-2.7	-3.5	**9.6**	**19.2**	-3.1	4.2	**9.3**	**9.3**	-0.7	2.5	-4.4	0.5	-4.8	-7.0	-4.0	0.2	-1.7
5 uM Simvastatin + 5 uM Cyclosporine	7.1	**15.5**	**-12.0**	-3.5	**16.1**	**-11.8**	**-11.4**	-5.9	-9.8	**-9.8**	**12.0**	**19.0**	**-4.9**	2.6	**10.4**	**7.6**	1.5	-0.5	-8.2	-2.9	-1.6	-5.7	-5.0	-0.3	**-3.2**

Figure S1. Twenty-five behavioral measures calculated using Z-LaP Tracker: The behavioral changes associated with 30 experimental treatments when compared to a DMSO control. Each value represents the percentage point difference between the active treatment and DMSO control. A 10-percentage-point increase in a behavioral measure is illustrated by red boxes, and a 10-percentage-point decrease by green boxes. Significant changes in a behavior parameter are denoted by a bolded and boxed cell ($p < 1.67 \times 10^{-3}$, correction for multiple comparisons [0.05/30]). Over 2300 larvae were tested, and the smallest n per arm was 30.
